# Supplementary material for: The Fly Homologue of MFSD11 Is Possibly Linked to Nutrient Homeostasis and Has a Potential Role in Locomotion: A First Characterization of the Atypical Solute Carrier CG18549 in Drosophila Melanogaster
Source: Insects. 2021 Nov 13;12(11):1024. doi: 10.3390/insects12111024 (PMC8621210; doi:10.3390/insects12111024)
Supplement: Supplementary file 1 [file insects-12-01024-s001.zip › Supplementary_Material.pdf]

Supplementary Material

1.1 S1 Appendix (Separate file)

Sequencing libraries were prepared from 1μg total RNA using the TruSeq stranded mRNA library preparation including polyA selection, and Illumina sequencing was performed. The sample files can be found at the SRA (PRJNA689052). The reads were mapped against the D. melanogaster genome assembly 6.09 using STAR mapper and was mapped against a genome index generated with the FlyBase GTF annotating file for the 6.09 genome assembly to direct mapping toward annotated genes. A total of 326,643,821 transcripts were mapped uniquely and used in subsequent analysis. The assembled transcripts were used in DESeq2 to obtain a final transcriptome assembly and to calculate the relative and differential expression. Genes were considered to be differentially expressed if they were significantly different (log2 fold change > 1 or <−1) in CG18549 knockdown flies compared with both controls in the DESeq2 analysis. ANOVA with FDR correction was used to identify expression differences between all three sample groups, and Wald test with Benjamini and Hochberg multiple correction method was used as post-hoc test.

1.2 Supplementary fig S1

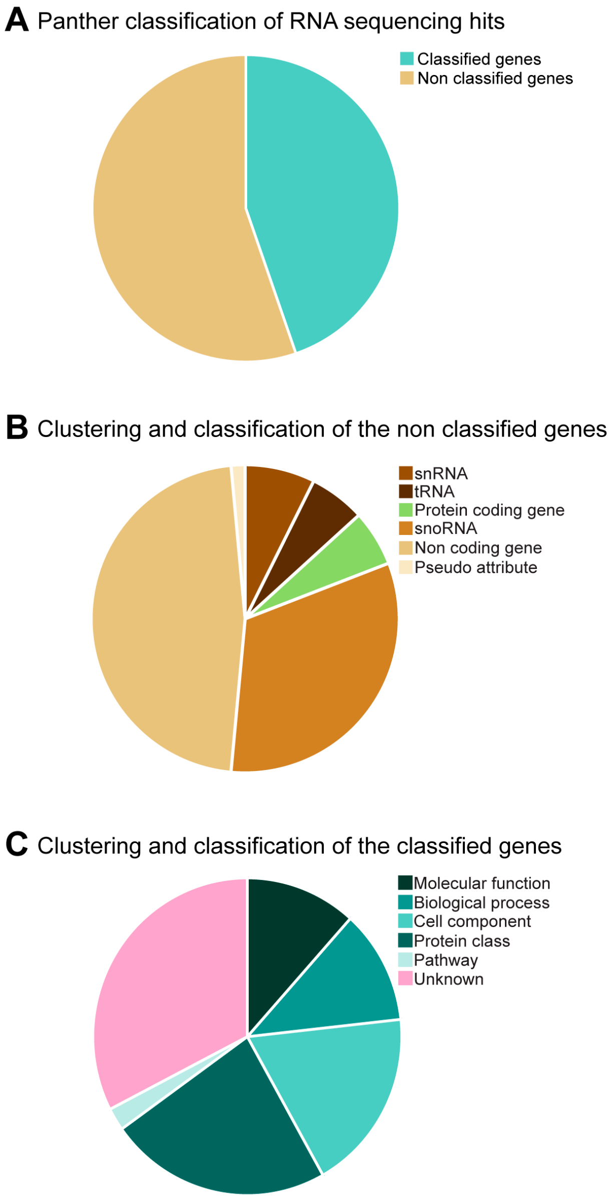

Figure S1. Protein ANalysis THrough Evolutionary Relationships (PANTHER) classification of RNA sequencing data. The assembled transcripts obtained from the RNA sequencing were used in DESeq2 to obtain a final transcriptome assembly and to calculate the relative and differential expression. Genes were considered to be differentially expressed if they were significantly different (log2 fold change > 2 or <−2) in *CG18549* KD flies compared with both controls in the DESeq2 analysis. The PANTHER (Protein ANalysis THrough Evolutionary Relationships) Classification System was used to investigate clustering and pathway mapping. In total 17, 560 unique genes were mapped (Supplementary material, Supplementary data 1), of those 125 genes were found to be affected in CG18549 knockdown flies. (A) 56 % of the genes could not be and 44 % of the genes could be classified according to PANTHER. (B) The majority of the non-classified genes were genes with pseudo attributes, snRNA, snoRNA and tRNA, as well as four protein coding genes. (C) The 55 genes that could be classified using PANTHER was clustered into six different categories:

biological process, cell component, molecular function, protein class, pathway and unknown, where the unknown (or “undefined) cluster was the largest followed by the protein class cluster.
