# Supplementary figures and images for: The Fly Homologue of MFSD11 Is Possibly Linked to Nutrient Homeostasis and Has a Potential Role in Locomotion: A First Characterization of the Atypical Solute Carrier CG18549 in Drosophila Melanogaster
Source: Insects. 2021 Nov 13;12(11):1024. doi: 10.3390/insects12111024 (PMC8621210; doi:10.3390/insects12111024)

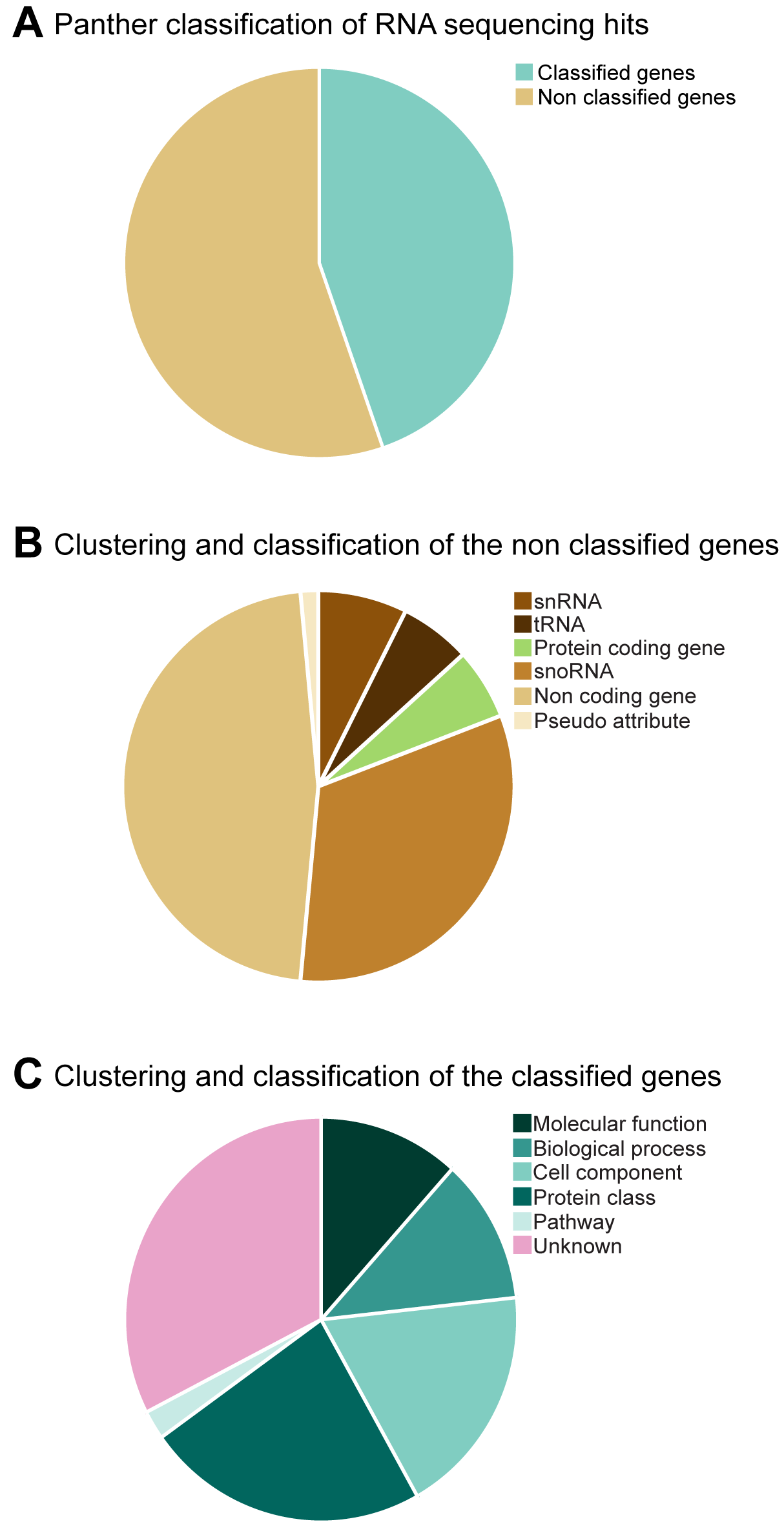

Supplement: Supplementary file 1 [file insects-12-01024-s001.zip › Supplementary Figure S1.tif]
